# Medical Lecture Season

**Published:** 1861-11

**Authors:** 


### EDITORIAL DEPARTMENT.

### MEDICAL LECTURE SEASON.

The present time offers inducements to young men commencing the study of medicine, hitherto unknown in the history of our country; instead of looking forward to years of waiting and watching, without business and without reward, an attractive field is now open to them as soon as they complete their professional course of study. Most of the well qualified young medical men who have desired it, have found ready employment and ample opportunity for improvement, in our Army and Navy, where they immediately receive compensation for their services, enjoy a wide field for observing surgical diseases and accidents, and at the same time obtain professional position and reputation, if from no other cause, from having passed successfully the examining board, which is generally believed to guard as faithful janitor, the door, to the medical staff, of the Army and Navy.

The season for Medical Lectures is at hand, and we see evidences in the unusually large class now attending Dissecting and Preliminary term, that others, who have not yet completed their medical education, are alive and active, fully appreciating the opportunity now offered, not only of obtaining their diploma, the *sine qua non*, without which, no young man can expect success or respectibility as a practitioner, but also of being installed immediately into active professional life, where his services will be demanded and appreciated.

Again we have no doubt the appointment of medical cadets, will have some influence in augmenting the size of the medical classes, since one term of lectures and two years study will make students eligible to this appointment, where young men will obtain many advantages and opportunities which cannot fail to stimulate those who have commenced the study of medicine. There are other influences now operating which will yet more sensibly increase the numbers of young men who will choose the profession of medicine for the business of life—I refer to that growing confidence which is placed in legitimate medicine over the various forms of irregular practice which have for the past few years received countenance, but now are becoming only a "hissing and a by-word" with sensible people, who are willing to know the truth.

While Military Surgery is opening a wide and attractive field for medical students and physicians, it is desirable, and proper, that medical schools should provide for ample instruction, in this most important department. The general principles of surgery must be applicable in all conditions and under all circumstances, still this will not supercede the necessity of teaching the peculiarities of military surgery in full detail, the most minute instructions in every thing relating to camp, military hospital, and field practice, cannot fail to be interesting and important, indeed indispensible, to the complete education of medical men at the present time.

In this connection and while speaking of its importance and necessity, we take great pleasure in saying, that in this respect the Faculty of the University of Buffalo, have made the most ample provision, and medical men as well as medical students, who propose to engage for a time in military practice, will be interested and instructed in all the essentials of medical military life, and we have no doubt that young physicians who are looking for appointment in the volunteer or regular service, will be most happy to avail themselves of this opportunity. In all our medical schools, doubtless attention will be given to this branch of education since the necessities of the country so imperatively demand it, but we do not hesitate in saying that instruction in this, as truly as in every other department, will be as full, comprehensive and practical, in the University of Buffalo, as in any institution in our country.

This struggle for the maintenance of the best government that was ever instituted on earth, though in many respects oppressive and painful, yet to the medical observer, and in a medical point of view, it presents some of the most pleasant prospects, and gives ground for bright hopes for the future of our profession. We expect in its final issues, aside from political conditions and influences, an advancement in civilization ; we expect Americans will learn a truer estimate of real worth, and a heartier contempt for barren pretention ; we expect a prize to be offered, not only for loyalty, but for competence and real merit, while a rebuke is given to political rebellion, and medical quackery. By this war we hope, and expect, these two things will be effectually squelched. Indeed, the exclusion of our young quacks from all public position, in the Army, Navy, and elsewhere, has already done much to weaken their faith in the "unknown Gods."

We most heartily congratulate those about to enter the profession upon the "times in which they have fallen"—at length not only those who "minister at the altar," of imposition and deception, but the masses, also, are throwing away their idols, and returning to regard, and appreciate, the regular profession ; better inducements are offered to ambitious, well qualified young men, to enter the profession of medicine; the night of darkness, superstition and ignorance has already in some degree passed away, and we think we see the dawning of a more glorious day for legitimate medicine.

